# Advisory Medical Boards for Insane Asylums

**Published:** 1864-11

**Authors:** 


### MISCELLANEOUS.

### ADVISORY MEDICAL BOARDS FOR INSANE ASYLUMS.

Communicated for the Boston Medical and Surgical Journal.

In the number of this Journal for October 13th, appeared a paper read before the Suffolk District Medical Society, and one of a series concerning "The Medical Management of Insane Women,' in which it is attempted to show the necessity and also the great utility of a Medical Board of Advisors to our Insane Asylums, for the benefit of the patients, the hospitals, the superintendents, the profession and the community.

The writer of that paper says, "his remarks are based upon extended observation, patient reflection," etc. It is quite possible if his observation could be a little further "*extended*" he would not only discover the uselessness of such a board, but the positive disadvantages of adding any more official boards to our already liberal allowance of disinterested guardians of the public charitable institutions for the insane. He is not aware, it seems, of the privilege granted superintendents by the trustees, and expressly and wisely directed sometimes by the by-laws of our hospitals, to call in consultation at any time such physician or surgeon as they may think proper for special cases. It would seem to be much more in accordance with an intelligent understanding of the whole subject, to allow the superintendent, who may be supposed to know what he needs, to choose his advisor from the whole range of his professional brethren, than to have him confined to a board appointed, as it might be, for personal or political reasons. In the latter case he is *compelled to take* such advice as the appointed board may be *able and willing* to give. If, as the writer allows, the advisory board visits patients only at the request of the superintendents (and no other course would be tolerated by any competent superintendent,) it is not difficult to see where the real advantrge would be secured.

The writer of this article is familiar with the duties required of physicians in insane asylums, having acted both as assistant and superintendent in one of the most important of our New England hospitals; and while so employed he had occasion to call consultations occasionally, and he can cheerfully testify of the aid and comfort so received. But in no instance did he feel the need of any *permanent* board of supervisors, *such as is recommended.*

It is said above that the advisory board might be appointed for political reasons. It might not. But by what influence was the late board of "Commissioners in Insanity" selected? For what reason were all the well-known "experts," able men and true, omitted from that Board? Without any wish to intimate that said Board was deficient, either in talent, experience or accomplishment for the task required, it is sufficient to say that of the three constituting that Board, two only were physicians, that neither of these was ever heard of as an "expert" in insanity, or as being inside the walls of an insane asylum in any other capacity than as an accidental or curious visitor, before his appointment to examine and report upon the difficult and complicated subject of insanity.

But let us examine some of the points taken by the author of the paper referred to. 1st. The advantage of an advisory board to the patients.

He has not made it very clear how the *mere appointment* of an advisory physician presents any advantage over the existing arrangement, when he admits that said advisor shall be called upon only at the discretion of the superintendent. The difference would consist merely in the person thought fit by the Governor, or whoever had the power of appointment, to give advice, and the person thought by the superintendent, a medical man and capable of judging of all the circumstances as they may arise, most suitable to aid him in any particular case.

If patients are not treated "*secundum peritissimam artem*" in any hospital at the present day, that hospital is under the care of an incompetent medical superintendent, and the fault is in the man, and not in the want of a board of advisors who, if appointed, could not act unless *called upon.* And the testimony of the quotation from the letter of one superintendent, saying "he could not make such an examination as was necessary for a full understanding of the case," etc., is "conclusive" only of the lack of a *disposition* on his part to *make use* of all the means of treatment considered proper to aid in restoring his patient.

In regard to the risks to which superintendents are liable from the delusions of insane patients, an advisory board would not relieve them except to give a wider field for the speculator. The danger is just as great every day, and when nothing out of the usual order of practice is said or done, if *delusions* are sufficient to annoy or victimize the managers of insane asylums.

Of the advantages of an advisory board to the profession.

The establishment of advisory boards *would*, undoubtedly, "stimulate physicians to more frequent study of mental disease," and it would afford a larger number from which to select gentlemen for superintendents and trustees. So far there would be an apparent advantage. But if the holding of such an office should lead them to suppose themselves qualified thereby to take the charge of one of our hospitals containing three or four hundred unsound minds, the *disadvantage*, after a very short time, would be *very* apparent.

As an example of the feeling that might be engendered by holding such an office, let us quote a few lines from the paper *under treatment:*

"As a single instance of what would naturally and generally be the result, it may be mentioned that shortly after the report of the late Massachusetts Commission in Insanity had been rendered, it was urged upon two of the three gentlemen constituting that Board, that they should allow their own names to be used as eligible to the vacant superintendency of the Northampton Asylum. Upon the third of their number similar arguments would have been used had he been a physician."

If either of the gentlemen *so urged* supposed for a moment that by yielding to the advice of his partial but imprudent friends he would have secured the countenance and support of a single member of the intelligent Board who had the power of appointment, he is certainly under a great delusion.

This opinion is based upon *extended observation*, and given for the benefit of any who may hereafter entertain similar sentiments, holding office bringing them slightly in relation to the subject of insanity. *

